# Supplementary material for: IgA autoantibodies promote inflammation, Th17 polarization and fibrotic responses in hidradenitis suppurativa
Source: Nat Commun. 2026 Mar 24;17:4469. doi: 10.1038/s41467-026-70883-5 (PMC13187259; doi:10.1038/s41467-026-70883-5)
Supplement: Supplementary file 1 — Supplementary Information [file 41467_2026_70883_MOESM1_ESM.pdf]

IgA autoantibodies promote inflammation, Th17 polarization and fibrotic responses in hidradenitis suppurativa

Carmelo Carmona-Rivera[1*], Liam J. O'Neil[2], Eduardo Patino-Martinez[1], William G. Ambler[1], Teja Mallela[3], Norio Hanata[1], Arsema K Zadu[4], Kan Jiang[5], Ginette A. Okoye[4,6], Angel S. Byrd[4,6], Christopher J. Sayed[3], Mariana J. Kaplan[1]

Supplementary Figures

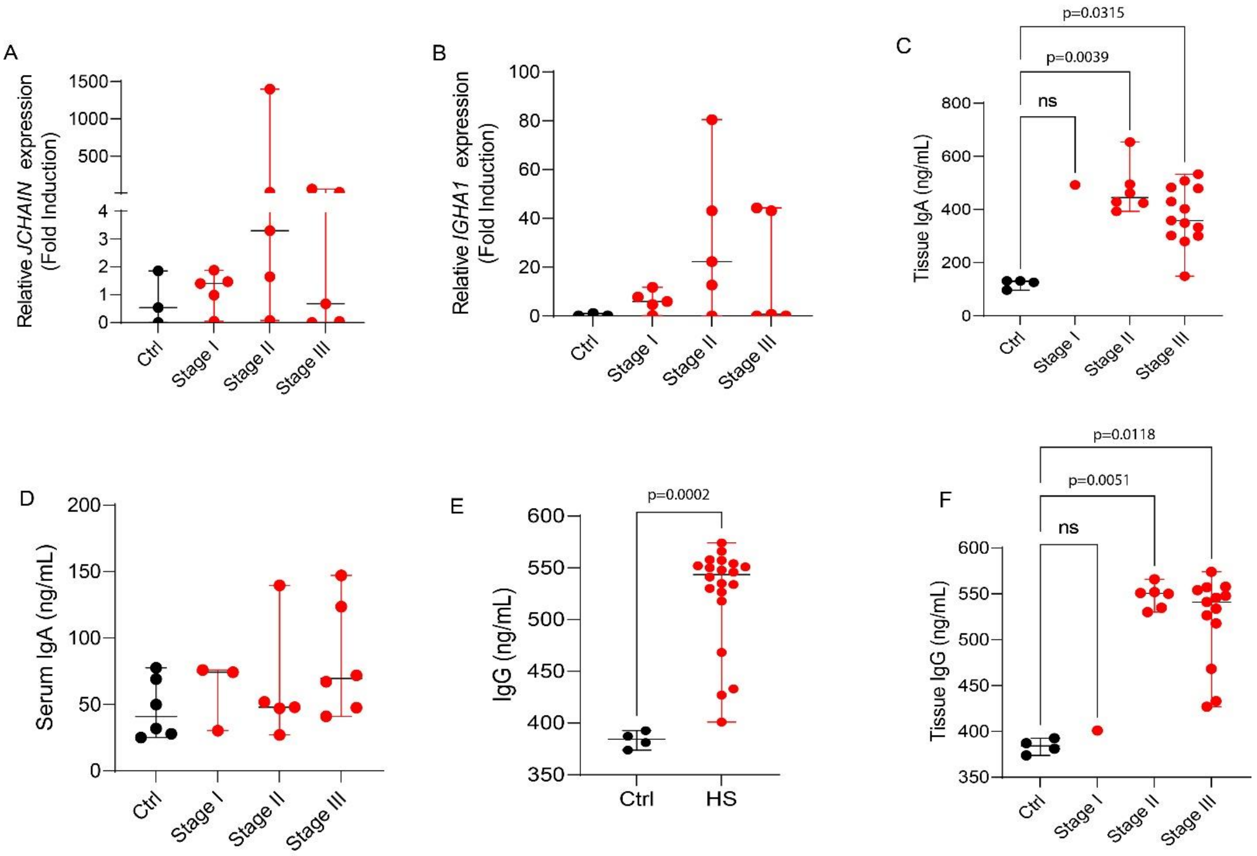

Supplementary Figure S1. (A) qPCR analysis of *JCHAIN* and (B) *IGHA1* expression in HS patients (stage I: n = 5; stage II: n = 5; stage III: n = 5) compared to control tissue (Ctrl, n = 3); (C-D) ELISA quantification of total IgA levels in HS patients (stage I: n = 1-3; stage II: n = 5-6; stage III: n = 6-14) versus control skin and serum samples. (E-F) Skin lysates from controls (n = 4) and HS patients n=20 (stage I: n = 1; stage II: n = 5; stage III: n = 14) were analyzed for total IgG. Results are the median ± range of three independent experiments in duplicate in A-F. For the

statistical analysis, 2-sided unpaired Mann-Whitney U-test was used in E and Kruskal-Wallis test was used in A-D and F.

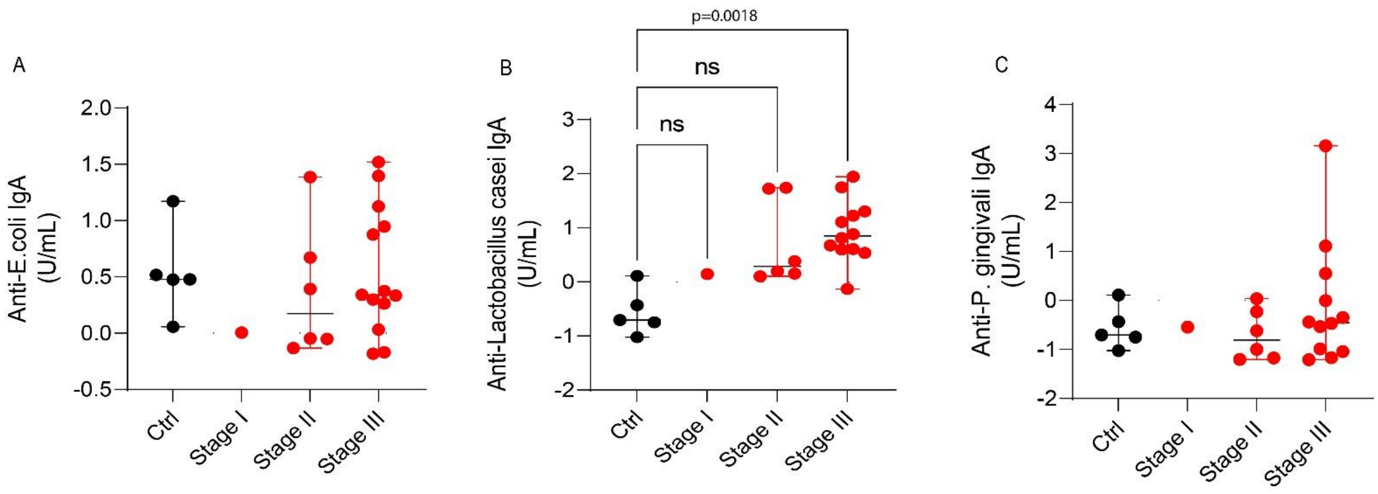

Supplementary Figure S2. Skin lysates from controls (n = 4) and HS patients (stage I: n = 1; stage II: n = 5; stage III: n = 14) were analyzed for IgA antibodies against (A) *E. coli*, (B) *Lactobacillus casei*, and (C) *P. gingivalis.* Results are the median ± range of three independent experiments in duplicate. Statistical significance was assessed using the Kruskal-Wallis test in A-C.

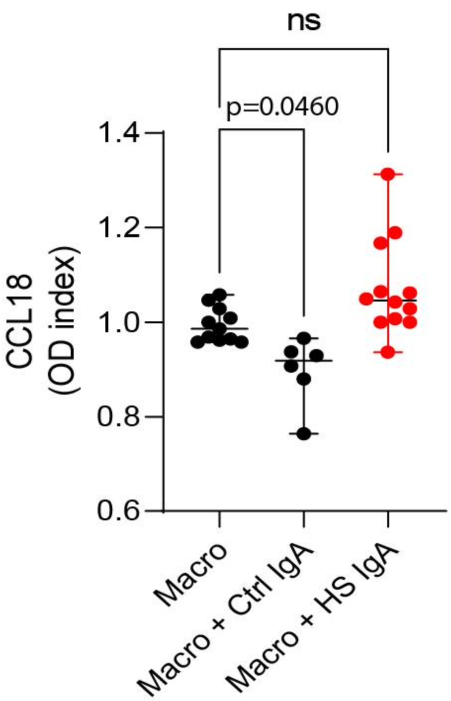

Supplementary Figure S3. Levels of CCL18 in M2 macrophages treated with purified IgA from healthy control (n=6) or HS skin tissue (n=12). Results are the median ± range of six independent experiments in duplicate. Statistical significance was assessed using the Kruskal-Wallis test.
